# A Mysterious Case of Recurrent Acute Hyperammonemic Encephalopathy

**DOI:** 10.7759/cureus.7484

**Published:** 2020-03-31

**Authors:** Venkata Satish Pendela, Pujitha Kudaravalli, Anisleidys Munoz, Gaby Razzouk

**Affiliations:** 1 Internal Medicine, Rochester General Hospital, Rochester, USA; 2 Internal Medicine, State University of New York (SUNY) Upstate Medical University, Syracuse, USA

**Keywords:** non-cirrhotic hyperammonemia, urea cycle disorders, altered mental status

## Abstract

Ammonia is a well-recognized neurotoxin. Awareness about hyperammonemia, in the absence of liver cirrhosis, may help in lifesaving, prompt diagnosis, and treatment. We present a case of a 53-year-old male who presented to the emergency department (ED) with altered mental status (AMS). He was unresponsive with occasional eye opening. Initial labs were normal except for mildly elevated blood alcohol level. Serum ammonia levels were very high (305 umol/L). He improved with lactulose. He had similar admissions later on. Urine orotic acid levels were high confirming ornithine transcarbamylase (OTC) deficiency. Noncirrhotic hyperammonemia as a cause of AMS remains a diagnosis of exclusion requiring high index suspicion. Very few cases of late inborn errors of urea cycle disorders (UCDs) have been reported in the literature. Our case highlights the importance of early diagnosis of UCDs and that outcome can be excellent if treated aggressively. Once identified, adult-onset forms of the UCDs have a good prognosis-largely due to the initiation of preventative measures and earlier recognition of exacerbations.

## Introduction

Confusion and altered mental status (AMS) are seen in 2% of the patients visiting the emergency department [1]. Ammonia is a well-recognized neurotoxin, mostly implicated in hepatic encephalopathy (HE) [2,3]. HE is one of the major causes of toxic metabolic encephalopathies which can result in acute emergency department (ED) visits. Hyperammonemia is related to severe liver disease in 90% cases, the remaining 10% is constituted by other conditions leading to disturbances in its excretion or production [2]. Awareness about hyperammonemia, in the absence of severe hepatic disease, may help in lifesaving, prompt diagnosis, and treatment.

## Case presentation

A 53-year-old African-American male was brought to the ED after his family members found him unresponsive. The family did not notice any alcohol, needles or empty pill bottles inside the house. His past medical history was significant for exercise controlled hypertension. He had no known allergies. On examination, he was unconscious with occasional spontaneous eye-opening and left gaze preference. Heart rate was 75 beats per minute, blood pressure 130/70 mmHg, temperature 99 °F, and respiratory rate 10 per minute. Neurological exam did not reveal neck rigidity or altered tone. Abdomen exam was benign.

Computed tomography (CT) of the head and CT angiogram of the neck did not reveal any acute abnormalities. Initial blood work revealed normal hemoglobin (14 g/dl), serum sodium (139 meq/L), arterial blood gas analysis, liver enzymes, and bilirubin. Serum ammonia level was elevated at 305 umol/L (Table 1).

**Table 1:** Investigations TSH- thyroid stimulating hormone, AST- aspartate aminotransferase, ALT- alanine aminotransferase, MRI- magnetic resonance imaging, CT- computed tomography

Investigation	Result
White cell counts	5.5×10^9^/L
Serum sodium	139meq/L
Blood urea nitrogen	6mg/dl
Urine drug toxicology	Negative
TSH	0.56 uIU/ml
Serum salicylate	<3 mg/dl
Serum Acetaminophen level	<10µg/ml
Blood alcohol	0.089 g/dl%
AST	40 U/L
ALT	26 U/L
Total bilirubin	0.4 mg/dl
Serum Ammonia	305umol/L
MRI brain	Normal study
Ultrasound abdomen	No evidence of liver cirrhosis
CT abdomen	Normal liver contour
CT head and neck	No acute abnormalities

**Figure 1:**
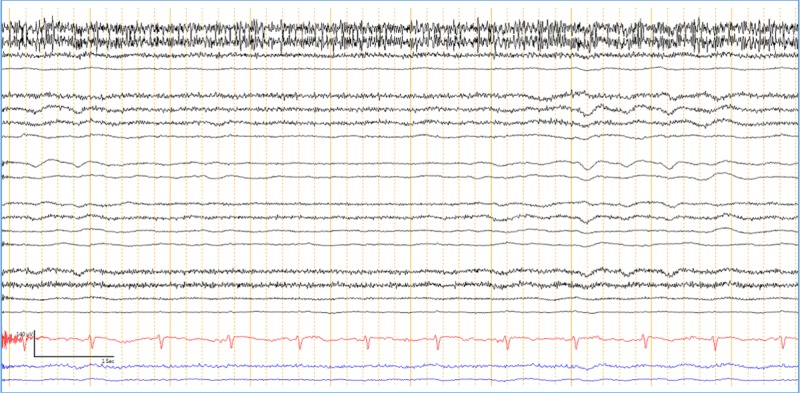
Electroencephalogram (EEG) showing diffuse encephalopathy

An electroencephalogram (EEG) revealed diffuse encephalopathy (Figure 1).

**Figure 2:**
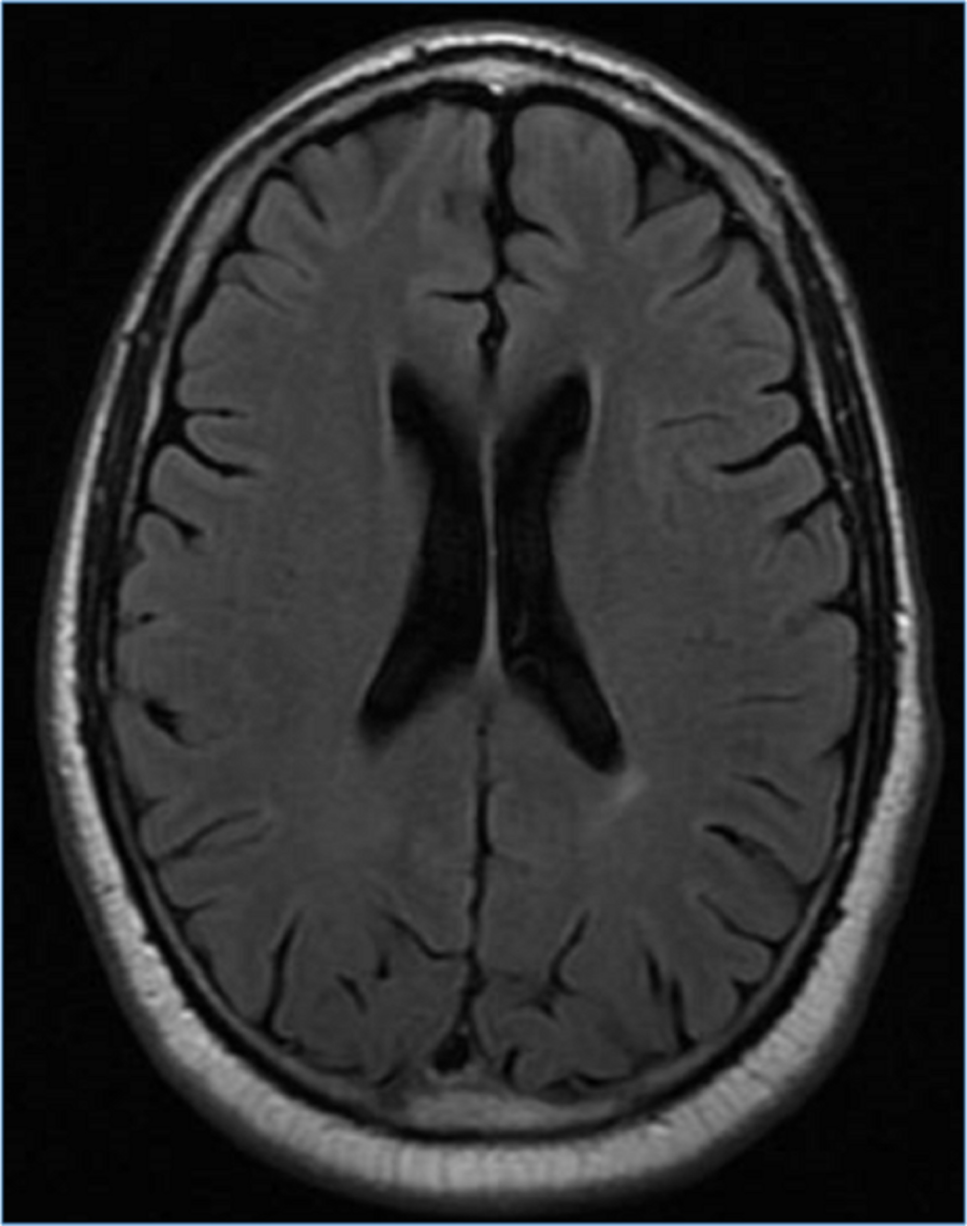
Magnetic resonance imaging (MRI) of the brain did not reveal any acute abnormality

Magnetic resonance imaging (MRI) of the brain did not show any acute abnormality (Figure 2).

A nasogastric tube was inserted and lactulose (30 g thrice daily) was administered. By the next day, he regained consciousness, and the serum ammonia decreased to 25 umol/L. Ultrasound liver and CT abdomen ruled out cirrhosis. He was discharged with a prescription of lactulose.

He returned to the ED with another episode of unresponsiveness a month later and was found to have elevated serum ammonia of 213 umol/L. He was not compliant with lactulose and he continued to have similar episodes of hyperammonemic encephalopathy. Serum amino acid levels were normal. Orotic acid in urine was high (>200 mmol/mol creatinine), clinching the diagnosis of ornithine transcarbamylase (OTC) deficiency. He was advised to consume a low protein diet and was prescribed L-carnitine along with lactulose.

## Discussion

Ammonia directly affects neuronal electric activity by inhibiting the generation of both excitatory and inhibitory postsynaptic potentials. In a healthy human, the ammonia produced by protein digestion is converted into urea by the peri-portal hepatocytes, protecting the brain from the adversities of ammonia [4].

**Figure 3:**
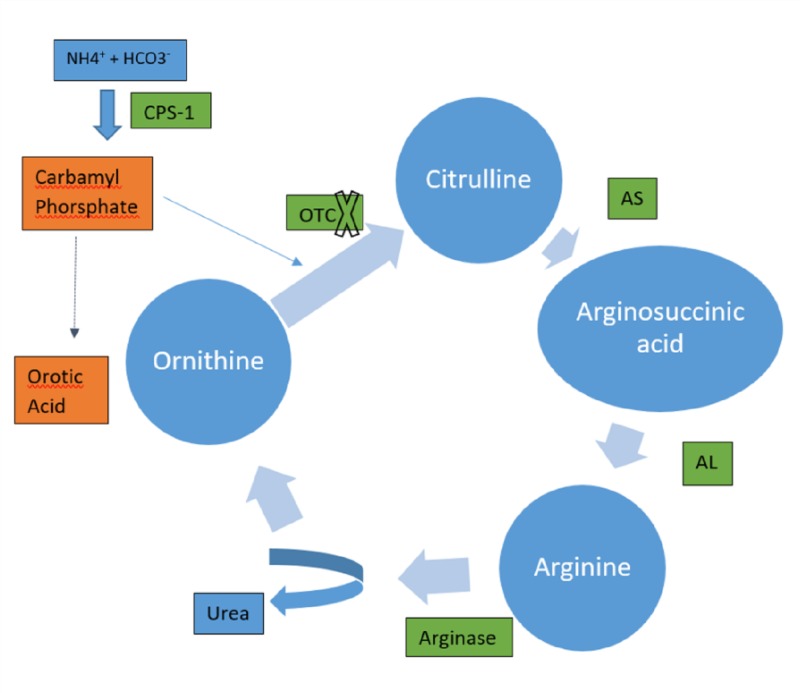
Urea cycle OTC deficiency leads to increased orotic acid excretion. AS- argininosuccinate synthetase, AL- Argininosuccinate lyase, CPS- carbamoyl phosphate synthetase I, OTC- ornithine transcarbamylase.

Secondary hyperammonemia occurs commonly in the presence of hepatic disorders leading to porto-systemic shunting. Less common causes are described in Table 2. Primary hyperammonemia is a rare entity in adults and causes include milder forms of urea cycle disorders (UCD) like OTC, argininosuccinate lyase, etc. (Figure 3) (Table 2) [5-11].

**Table 2:** Various causes of hyperammonemia without chronic liver disease

Causes of non-hepatic hyperammonemia
Urea cycle disorders (Primary Hyperammonemia) like Carbamoyl phosphate synthase I deficiency, Ornithine transcarbamoylase (OTC) deficiency etc.
Secondary Hyperammonemia
Portosystemic shunts
Urinary diversion- ureterosigmoidostomy with Ileal conduit
Organic acidemias
Reye Syndrome
Drugs including Antiepileptics (valproate, carbamazepine), Anti-cancer drugs (5 fluorouracil)
Metabolic disorders like Fatty acid oxidation defects, Amino acid transport defects
Thyroid disease - Hashimoto’s encephalopathy
Hematological disorders including Multiple myeloma and Acute myeloid leukemia
Post gastric bypass surgery

OTC deﬁciency is the most common inherited UCD. It is diagnosed by the high level of urinary orotic acid, secondary to the diversion of carbamoyl phosphate via the cytosolic pyrimidine synthetic pathway [12]. Even though it is more common in children (neonatal period), milder forms (homozygous males and heterozygous females) can present directly in adulthood [13,14]. Hyperammonemia can present as personality changes, sleep-wake cycle alterations, seizures, and confusion. Untreated acute severe hyperammonemia (ammonia levels >200 umol/L) can lead to comatose state as well as death [15]. This is due to severe cerebral edema and raised intracranial pressure. Cytotoxic and vasogenic oedema mechanisms have been implicated.

Isolated hyperammonemia without disturbances in other liver functions should prompt further investigations. A blood gas analysis showing acidosis points towards organic acidemias and respiratory alkalosis should raise suspicion of UCD. Classic magnetic resonance (MR) findings include the involvement of the insular cortex and gyrus for unknown reasons. As seen in our patient, MRI could be normal. Acute treatment is targeted towards ammonia lowering. Non-absorbable disaccharides (lactulose) help in decreasing ammonia production as well as absorption from the intestines [16]. Antibiotics like rifaximin and neomycin are approved for hepatic encephalopathy with hyperammonemia [17]. However, neomycin has serious adverse effects like ototoxicity, neurotoxicity, and nephrotoxicity limiting its use. Intra-cranial pressure lowering with mannitol or hypertonic saline is administered in patients with brain edema, seizures or coma. Hemodialysis is used in refractory hyperammonemia patients. Long-term dietary modifications with higher calorie to nitrogen ratio is preferred for UCDs. L-carnitine supplement helps in lowering the frequency of hyperammonemic attacks [18-20]. Liver transplant is an important treatment modality in patients with recurrent acute severe encephalopathy in UCDs. 

The present work has been presented at the American College of Gastroenterology Conference 2019 [11].

(Abstract: Pendela VS, Munoz A, Razzouk G. A Mysterious Case of Non-Cirrhotic Recurrent Severe Hyperammonemia: What Urea-lly Need to Know. 2019 ACG Annual Meeting; October 2019).

## Conclusions

Very few cases of late inborn errors of UCDs have been reported in the literature. The majority of these cases are fatal and present in previously healthy adults. Noncirrhotic hyperammonemia as a cause of AMS remains a diagnosis of exclusion and requires high index suspicion. Our case highlights the importance of early diagnosis of UCDs and that outcome can be excellent if treated aggressively. Once identified, adult-onset forms of the UCDs have a good prognosis largely due to the initiation of preventative measures and earlier recognition of exacerbations.
